# SSR marker development in *Clerodendrum trichotomum* using transcriptome sequencing

**DOI:** 10.1371/journal.pone.0225451

**Published:** 2019-11-20

**Authors:** Gongwei Chen, Yuanzheng Yue, Yajie Hua, Die Hu, Tingting Shi, Zhaojing Chang, Xiulian Yang, Lianggui Wang

**Affiliations:** 1 College of Landscape Architecture, Nanjing Forestry University, Nanjing, Jiangsu, China; 2 College of Forestry, Nanjing Forestry University, Nanjing, Jiangsu, China; ICAR - National Research Center on Plant Biotechnology, INDIA

## Abstract

*Clerodendrum trichotomum*, a member of the Lamiaceae (Verbenaceae) family, is an ornamental plant widely distributed in South Asia. Previous studies have focused primarily on its growth characteristics, stress resistance, and pharmacological applications; however, molecular investigations remain limited. Considering germplasm conservation and the extensive applications of this plant, it is necessary to explore transcriptome resources and SSR makers for *C*. *trichotomum*. In the present study, RNA sequencing was used to determine the transcriptome of *C*. *trichotomum*. Subsequently, unigene annotations and classifications were obtained, and SSRs were mined with MIcroSAtellite. Finally, primer pairs designed with Oligo 6.0 were selected for polymorphism validation. In total, 127,325,666 high-quality reads were obtained, and 58,345 non-redundant unigenes were generated, of which 36,900 (63.24%) were annotated. Among the annotated unigenes, 35,980 (97.51%) had significant similarity to 607 species in Nr databases. In addition, a total of 6,444 SSRs were identified in 5,530 unigenes, and 200 random primer pairs were designed for polymorphism validation. Furthermore, after primary polymorphism identification, 30 polymorphic primer pairs were selected for the further polymorphism screening, and 200 alleles were identified, 197 of which showed polymorphism. In this work, a large number of unigenes were generated, and numerous SSRs were detected. These findings should be beneficial for further investigations into germplasm conservation and various applications of *C*. *trichotomum*. These results should also provide a solid foundation for future molecular biology studies in *C*. *trichotomum*.

## Introduction

*Clerodendrum trichotomum*, known as ‘Chou-Wu-Tong’ in China, is an ornamental plant found in wild areas with a temperate climate in China, Japan, Korea, and the Philippines [1]. As a widespread broad-leaved understory shrub, *C*. *trichotomum* is distributed primarily in thickets near hillsides, riversides, and roadsides below an elevation of 2400 m [2]. It is pollinated nocturnally and diurnally by animals, including hawkmoths, bees, and swallowtails [3]. In addition, *C*. *trichotomum* has been reported as a tree species with a strong capability to adapt to environmental challenges such as drought, barren and salt resistance, and therefore, it could be used for ecological restoration in mining areas and for afforestation in saline-alkali land [3]. *Clerodendrum*, a genus of the Lamiaceae (Verbenaceae) family, includes approximately 500 species distributed worldwide. Most plants in this genus, such as *C*. *chinense* [4], *C*. *indicum* [5], *C*. *izuinsulare* [6], *C*. *petasites* [7], *C*. *phlomidis* [8], and *C*. *trichotomum* [9], have pharmacological activities. In *C*. *trichotomum*, the roots, stems, and leaves have diverse pharmacological activities, including analgesic, anti-inflammatory, and sedative effects [10, 11].

Studies on *C*. *trichotomum* have mainly focused on growth characteristics, stress resistance, chemical components, and pharmacological applications [12]. Emphasis has been placed on the physiological biochemistry characteristics, as well as practical applications [13, 14]. However, studies on molecular mechanisms are limited [15], and understanding the molecular mechanisms underlying chemical-constituent biosynthesis, stress resistance, and growth characteristics will be important for the future development of pharmacological utility, ecology restoration, and cultivation. Transcriptome sequencing would be beneficial for studying these mechanisms, especially for exploring the influences of genes in relevant biologically complicated processes [16].

New technological advancements have broadened the range of DNA polymorphism assays for genetic mapping, genome fingerprinting, marker assisted plant breeding, and investigations of genetic relatedness [17]. These common techniques include analysis of amplified fragment length polymorphisms (AFLP), random amplified polymorphic DNA markers (RAPD), restriction fragment length polymorphisms (RFLP), and simple sequence repeat (SSR) microsatellites [18]. These methods detect polymorphisms by assaying subsets of the total DNA sequence variation within the genome [19]. Compared with the other three technologies, SSR methods have the highest expected heterozygosity. Polymorphisms based on SSR are likely to have a positive effect on plant genetics, because they enable highly informative PCR based assays that meet the requirements of plant breeding and population genetic programs [20].

With the improvement of next generation sequencing technology (NGS), RNA sequencing has gradually become a convenient and efficient technique for detecting SSR markers and has been extensively applied in numerous species [21, 22]. Given the limited genetic studies, SSRs would be the most abundant resources for genetic diversity analysis, cultivar identification and marker-assisted breeding of *C*. *trichotomum* [15]. Herein, RNA sequencing was conducted with the Illumina 4000^TM^ platform, and de novo assembly was performed, to facilitate genetic analysis and SSR marker detection through collection of sequence resources and genetic information. The transcriptomes and SSRs will be freely available to the public and should be helpful for *C*. *trichotomum* population genetics and phytochemical investigations, as well as breeding conservation.

## Materials and methods

### Plant materials, RNA isolation, and DNA extraction

The materials from 20 natural *C*. *trichotomum* populations were gathered from nine provinces, including most of the natural habitats in China (Table 1). Each tree for sampling was an autochthonous, healthy adult individual from a mountain forest or from a wayside of a primary forest in some cases. All trees used for sampling were at least 100 m apart from any dwelling district or cultivated farmland, and at least 20 m apart from each other.

**Table 1:** Information of *C*.*trichotomum* germplasm resources.

Germplasm resources	Population ID	Type	Sample Size	Longtitude(E)	Latitude(N)	Elevation (m)
1. Xuzhou, Jiangsu	XZ1	Wild	4	117°11 '06.80"	34°16 '33.82"	32
2. Xuzhou, Jiangsu	XZ2	Wild	4	117°10 '52.96"	34°13 '47.12"	44
3. Lianyungang, Jiangsu	LYG	Wild	4	119°18 '26.45"	34°37 '13.75"	56
4. Pingxiang, Jiangxi	PX	Wild	4	113°55 '57.56"	27°27 '33.92"	342
5. Jinhua, Zhejiang	JH	Wild	4	120°27 '12.03"	29°02 '58.60"	256
6. Tai’an, Shandong	TA	Wild	4	117°03 '51.93"	36°09 '37.12"	45
7. Pingdu, Shandong	PD	Wild	4	120°14 '02.69"	36°47 '32.53"	50
8. Dingzhou, Hebei	DZ	Wild	4	114°55 '29.66"	38°34 '51.90"	68
9. Jurong, Jiangsu	JR	Wild	4	119°18 '46.11"	32°04 '31.06"	47
10. Yangling, Shanxi	YL	Wild	4	108°24 '38.57"	34°16 '04.92"	418
11. Jinan, Shandong	JN	Wild	4	116°46 '15.26"	36°32 '39.13"	110
12. Feicheng, Shandong	FC	Wild	4	116°50 '42.11"	36°09 '58.04"	214
13. Mount Tai, Shandong	MT	Wild	4	117°09 '24.13"	36°15 '22.96"	286
14. Longnan, Jiangxi	LN	Wild	4	114°46 '29.60"	24°53 '32.90"	222
15. Bicheng, Shandong	BC	Wild	4	117°36 '03.66"	34°46 '23.57"	90
16.Mount Tianzhu, Anhui	MT	Wild	4	116°12 '39.52"	31°22 '35.91"	219
17. Luoyang, Henan	LY	Wild	4	111°03 '28.31"	34°02 '32.08"	606
18. Nanjing, Jiangsu	NJ	Wild	4	118°49 '38.49"	32°03 '41.23"	67
19. Guilin, Guangxi	GL	Wild	4	110°12 '19.89"	25°15 '47.19"	161
20. Zhengzhou, Henan	ZH	Wild	4	113°38 '48.67"	34°52 '15.47"	93

For transcriptome sequencing, fresh roots, stems, leaves, flower buds, petals, calyxes, and fruits of the Pingdu, Shandong (PD) population were collected on 10 August, 2016 from three single, mature, healthy-appearing *C*. *trichotomum* trees and immediately frozen in liquid nitrogen. Then, the samples were kept at -80°C in the Molecular Library in Nanjing Forestry University. Total RNA isolation was carried out with an RNAprep pure Kit (Tiangen, Beijing, China), according to the manufacturer’s instructions. RNA contamination and degradation were monitored with 1% agarose gels. RNA purity was assayed with a NanoDrop 2000 Spectrophotometer (Thermo Scientific, USA), and the sample integrity was tested with the Agilent 2100 Bioanalyzer system (Agilent Technologies, USA). Equivalent amounts of RNA from fresh root, stem, leaf, flower bud, petal, calyx, and fruit samples were pooled together for RNA sequencing.

To verify SSR polymorphisms, we extracted DNA from 80 leaf samples collected in August, 2016 from *C*. *trichotomum* trees of 20 wild populations in China (Table 1). The genomic DNA was extracted with a Plant Genomic DNA Kit (Tiangen, China), according to the manufacturer’s instructions. The purity of the extracted DNA was determined on a NanoDrop 2000 Spectrophotometer (Thermo Scientific, USA), the concentration was measured with a Qubit® Flurometer 2.0 (Life Technologies, USA), and the integrity was assessed with an Agilent 2100 Bioanalyzer system (Agilent Technologies, USA). The DNA samples were finally stored at -20°C after being diluted to 25 ng μL^-1^.

### RNA-seq library construction for sequencing

RNA-seq libraries were prepared with an NEBNext Ultra^TM^ RNA library Prep Kit for Illumina (NEB, Beverly, MA, USA), according to the manufacturer’s instructions. With Oligo (dT), poly (A)^+^ mRNA was isolated with beads after total RNA was obtained, and the mRNA was cut into short fragments with fragmentation buffer. Subsequently, with random primers and reverse transcriptase, first-strand cDNA was synthesized from the RNA fragments (Invitrogen, USA). Then, second-strand cDNA was synthesized with buffer, RNase H, DNA polymerase I, and dNTPs. After adapter ligation, a single ‘A’ base was added to the 3’ end for end repair of cDNA fragments. Through the process of amplification, cDNAs were separated on agarose gels, and the cDNA library was finally generated.

### RNA-seq and de novo assembly

Illumina HiSeq^TM^ 4000 sequencing was performed by Gene Denovo Biotechnology Co. (Guangzhou, China). For raw reads obtained by sequencing, filtration was performed to remove reads with adaptors, with more than 10% unknown nucleotides (N) or with more than 50% low quality (Q-value ≤ 10) bases. After filtration, high quality clean reads were obtained.

The transcriptome *de novo* assembly was achieved with Trinity short read assembly software (http://trinityrnaseq.sourceforge.net). First, reads were assembled into linear contigs through a k-mer based approach. Next, related contigs corresponding to portions of alternatively spliced transcripts or unique portions of paralogous genes were clustered. Subsequently, de Brujin graphs for clusters were constructed. Finally, the paths taken by reads in the corresponding de Brujin graph were analyzed. Then, transcripts derived from paralogous genes and linear sequences from each alternatively spliced isoform were obtained [23].

### Function annotation and classification of unigenes

To annotate and classify the transcriptome, a BLASTX search (E-value < 10^−5^) was performed against protein databases, such as NCBI non-redundant protein (Nr), Gene Ontology (GO), Clusters of Orthologous Groups (COG), and Kyoto Encyclopedia of Genes and Genomes (KEGG). Blast2GO (v2.5.0) was used to obtain GO annotations (E-value < 10^−5^) on the basis of the Nr notes. GO terms were plotted for categorization with Web Gene Ontology Annotation Plot (WEGO), and the unigenes were aligned for function prediction and classification according to the COG and KOG databases. Unigenes containing SSRs were also searched against the KOG database through BLASTX. KEGG pathways were used to distinguish the assembled sequences in the online software (www.genome.jp/kegg/kegg4.html).

### SSR prediction, primer design, and polymorphism identification

Microsatellites were detected within the unigenes longer than 1000 bp via MIcroSAtellite (MISA, http://pgrc.ipkgatersleben.de/misa/). SSRs with two to six repeat motifs and flanking sequences longer than 100 bp were considered. Compound SSRs (in which the interval within the SSRs was no more than 100 bp) were excluded.

According to the MISA results, we designed primers on the basis of the principle that the predicted product size was 100–300 bp and no introns were present. Oligo6.0 software was used to design primer pairs according to the following criteria: (1) content of GC between 40% and 60%; (2) amplified fragment length between 100 and 300 bp; (3) primer annealing temperature (Tm) between 57 and 62°C; (4) a difference between the annealing temperatures (Tm) of the forward and reverse primers within 3°C; (5) avoidance of primer dimers and hairpin structures. For other parameters, the default settings were used.

The primary primer polymorphism identification procedure was as follows: DNA samples from six wild populations, including PD, JR, LN, LY, GL and DZ, and 200 random primer pairs were used for the primary polymorphism identification; the Touch-down PCR was performed, and the procedure was as follows: initial denaturation for 4 min at 94°C, 30 s at 94°C, 45 s annealing at 65°C, 45 s renaturation for 10 cycles at 72°C, 30 s denaturation at 94°C, annealing temperature (Tm) for 45s, 30 s renaturation for 34 cycles at 72°C, and 10 min elongation at 72°C; primer polymorphisms were then identified with 8% polyacrylamide gel electrophoresis, and the successfully amplifying polymorphism primers were finally chosen for the further polymorphism identification in 20 *C*. *trichotomum* populations.

The total DNA of 80 individuals from 20 *C*. *trichotomum* populations was used for the further polymorphism identification. Fluorescence-labeled (FAM, HEX, TAMRA or ROX) capillary electrophoresis was used to accurately screen the variation among populations. PCR was performed in a 10-μL reaction system containing 3.0 mM MgCl2, 0.4 mM dNTPs (TaKaRa, Dalian, China), 0.2 U Taq polymerase (TaKaRa, Dalian, China), 0.6 μM forward primers, 0.6 μM reverse primers, and 40 ng of genomic DNA. The unified annealing temperature for PCR was 60°C, and the amplification conditions were the same as above. The successfully amplified products were then identified using an ABI 3730 DNA Analyzer (Applied Biosystems, Foster City, California, USA). Finally, Genemarker v2.2.0 was used to process the original data collected from the fluorescence-labeled capillary electrophoresis.

### Genetic diversity analysis of germplasm resources

Genetic diversity analysis was carried out in POPgene32 under the following parameters: number of alleles (Na), effective number of alleles (Ne), Nei’s diversity index (H), and Shannon’ information index (I). The polymorphic information content (PIC) was detected using PowerMarker. The genetic similarity coefficiency (GS) was calculated using NTSYS-PC software. Finally, the genetic clustering diagram of *C*.*trichotomum* was constructed by UPGMA method considering the genetic similarity coefficiency (GS).

## Results

### Raw read filtering and de novo assembly

A total of 128,455,668 raw reads were generated, and 127,325,666 high-quality clean reads remained after filtering; the Q20 reached 97.75%, and the GC content was approximately 46.38%. All transcriptome data has been deposited in the NCBI Sequence Read Archive under accession number SRP151646.

According to the results of *de novo* assembly, 58,345 unigenes were generated with an average length of 850 bp, GC content of 42.23%, N50 value of 1,525 bp, maximum length of 14,478 bp, and minimum length of 201 bp. The lengths of the unigenes were distributed as follows: 17,357 unigenes ≥ 1 kb, 5,942 unigenes ≥ 2 kb, and 1,756 unigenes ≥ 3 kb in length.

### Functional annotation

**Fig 1:**
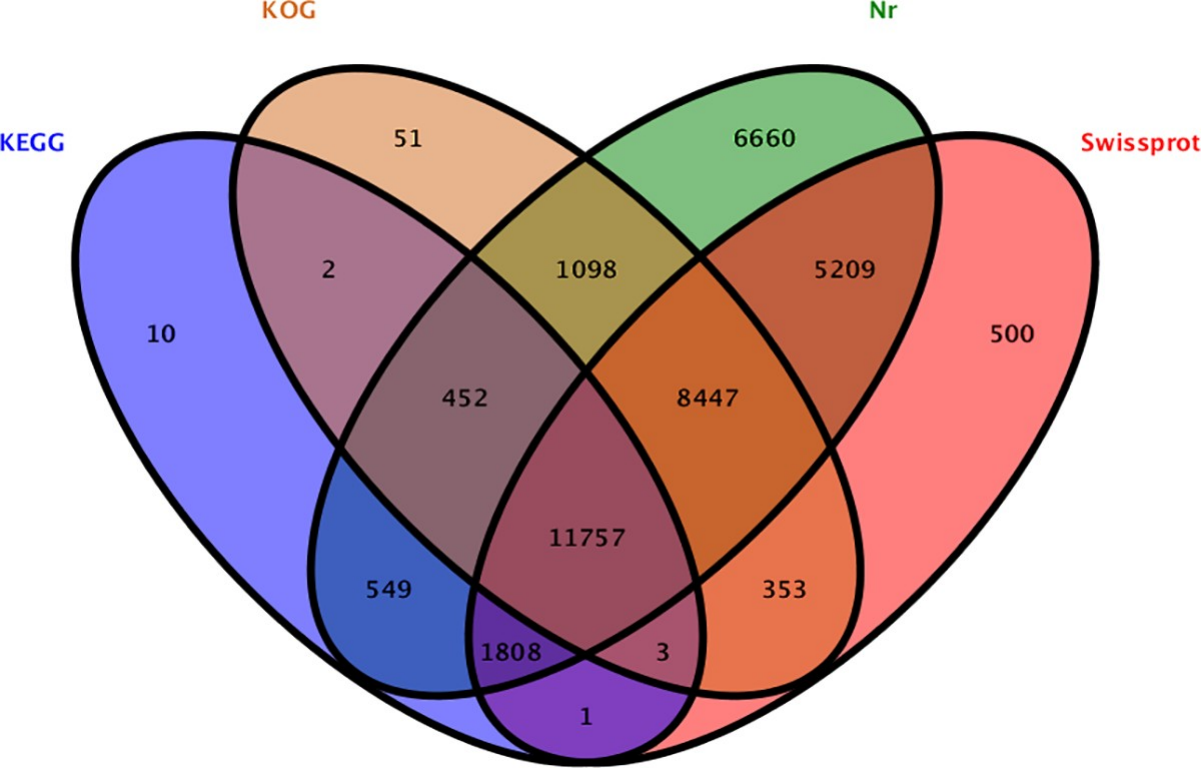
Venn diagram of annotation results.

Among the 58,345 identified unigenes, 36,900 (63.24%) were annotated according to relevant databases, 35,980 (97.51%) of which had significant matches in the Nr database, whereas 28,078 (76.09%), 22,163 (60.06%) and 14,582 (39.52%) of which had similarity to data in the Swiss-Prot, KOG, and KEGG databases, respectively. Among all the unigenes, 11,757 (31.86%) were successfully annotated in all the four above databases (Fig 1).

### Nr annotation

**Fig 2:**
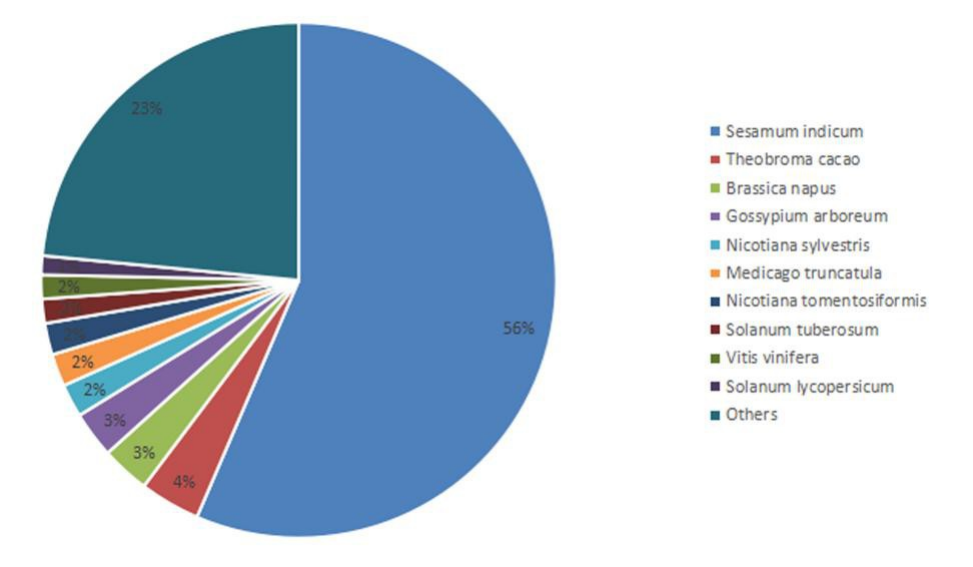
The Species distribution of Nr annotations.

In total, 35,980 unigenes were annotated from 607 popular model species in the Nr databases. The species distribution of Nr annotations included *Sesamum indicum* (20,319, 56.47%), *Theobroma cacao* (1,368, 3.80%), *Brassica napus* (1,092, 3.04%), *Gossypium arboreum* (1,047, 2.91%), *Nicotiana sylvestris* (749, 2.08%), *Medicago truncatula* (735, 2.04%), *Nicotiana tomentosiformis* (711, 1.98%), *Solanum tuberosum* (546, 1.52%), *Vitis vinifera* (541, 1.50%), *Solanum lycopersicum* (428, 1.19%), and others (Fig 2).

### GO classification

**Fig 3:**
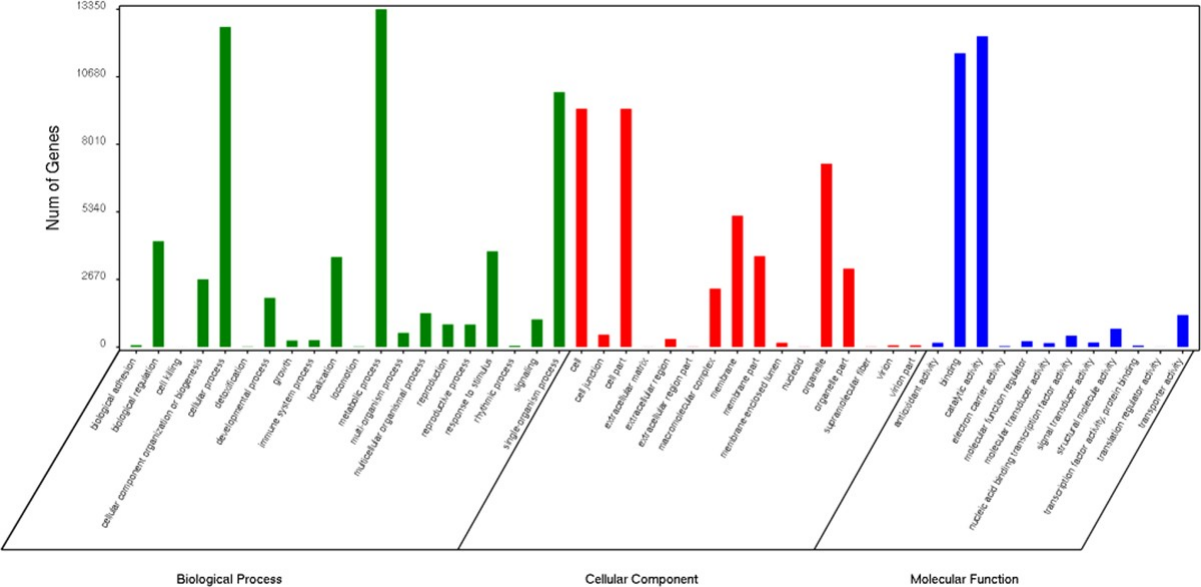
GO functional classification. Go functions are shown in the *X*-axis. The numbers of genes annotated with the GO function are shown in the *Y*-axis.

GO analysis was carried out with the categories of ‘biological process’, ‘cellular component’, and ‘molecular function’ (Fig 3). On the basis of the statistics, the unigenes could be subdivided into 20 terms in ‘biological process’, of which ‘metabolic process’, ‘cellular process’, and ‘single-organism process’ were highly represented. There were 16 ‘cellular component’ functional GO terms, in which ‘cell’ was the largest, followed by ‘cell part’ and ‘organelle’. In ‘molecular function’, there were 12 GO terms, among which ‘catalytic activity’, ‘binding’, and ‘transporter activity’ were the most abundant. These results macroscopically reflected the functional distribution characteristics of *C*. *trichotomum* unigenes.

### KOG classification

**Fig 4:**
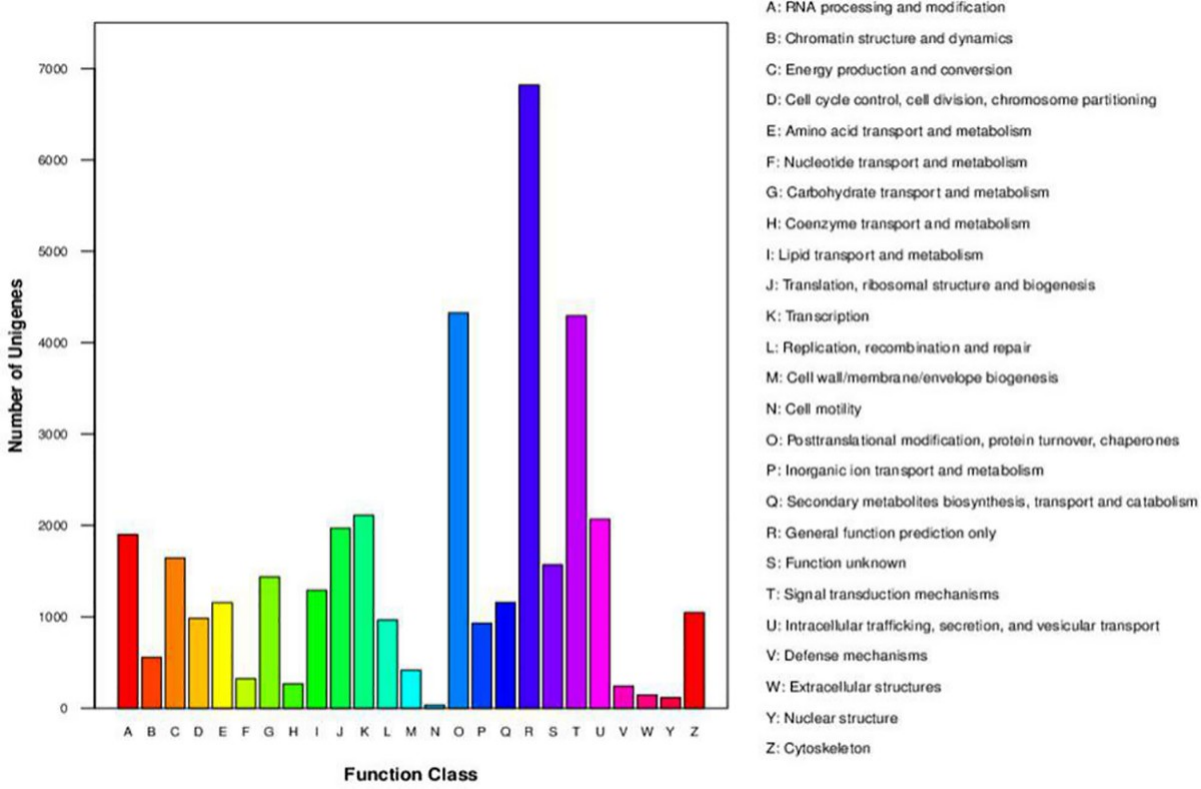
KOG fuction classification. The *X*-axis shows the functional classes of KOG, and the *Y*-axis shows the numbers of unigenes in every class.

A total of 58,345 unigenes were searched against the KOG database to obtain further functional prediction and classification, 22,163 of which were clustered into 25 functional categories (Fig 4). Among these categories, the largest cluster was R—‘general function prediction only’ (6,821, 30.78%)—followed by ‘posttranslational modification, protein turnover, chaperones’ (4,328, 19.53%), ‘signal transduction mechanisms’ (4,296, 19.38%), ‘transcription’ (2,114, 9.54%), and ‘intracellular trafficking, secretion, and vesicular transport’ (2,069, 9.34%). The smallest groups were ‘extracellular structures’ (144, 0.65%), ‘nuclear structures’ (117, 0.53%), and ‘cell motility’ (33, 0.15%). In addition, 1,571 (7.09%) unigenes were classified into cluster S—‘function unknown’.

### Transcription factor analysis

**Fig 5:**
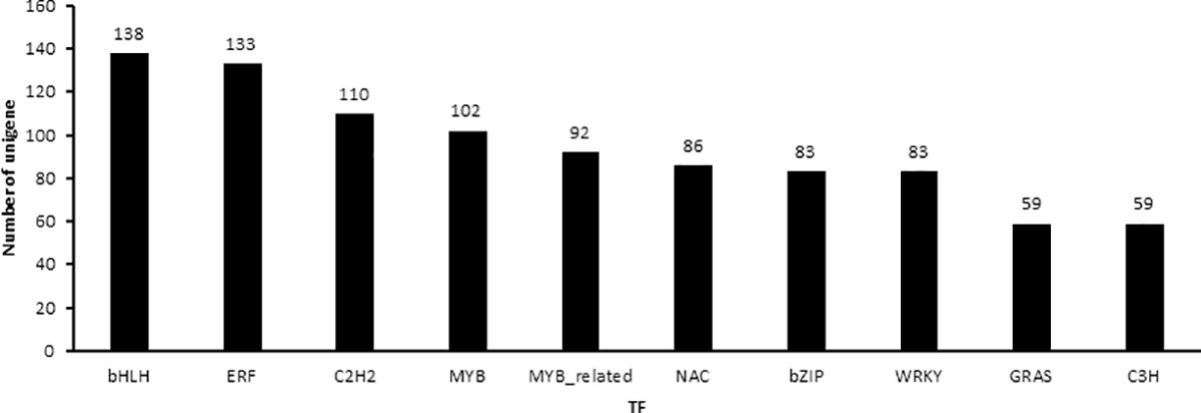
Main Type classification of predicted TF. The horizontal coordinates are the main type of TF, and the vertical coordinates are the numbers of unigene in one TF class.

In the present work, protein coding sequences of unigenes were aligned through BLASTp with Plant TFdb (http://planttfdb.cbi.pku.edu.cn/) to predict transcription factor (TF) families. Subsequently, 1,720 *C*. *trichotomum* unigenes were identified with predicted amino acid sequences matching 57 TF families (Fig 5). Among the TF families, ‘bHLH’ possessed the most unigenes (138), followed by ‘ERF’ (133), whereas ‘STAT’, ‘HB-PHD’, ‘NZZ/SPL’, and ‘HRT-like’ had only one each.

### Frequency and distribution of SSRs

MISA was used to identify SSR loci. All 58,345 assembled unigenes were used to mine potential SSRs, and a total of 5,530 unigenes containing 6,444 SSRs were identified. Among these unigenes containing SSRs, 781 unigenes contained more than one SSR, and 411 SSRs presented a compound formation. On average, one SSR was found every 7.70 kbp. Among the mined SSRs, dinucleotide motifs were the most enriched (4,112, 63.81%), followed by tri- (1,523, 23.63%), tetra- (441, 6.84%), penta- (234, 3.63%), and hexa- (134, 2.08%) nucleotide motifs (Table 2).

**Table 2:** Classification of SSR loci of *C*. *trichotomum*.

Repetition	Type of repeat motif					Total
	di	tri	tetra	penta	hexa	
4	0	0	337	112	149	598
5	0	935	84	13	61	1093
6	1362	337	14	7	9	1729
7	949	136	3	0	2	1090
8	699	28	1	0	8	736
9	495	11	1	0	2	509
10	323	22	0	0	0	345
11	155	4	0	1	1	161
12	31	13	1	0	1	46
13	4	8	0	0	0	12
14	5	7	0	0	1	13
> = 15	89	22	0	1	0	112
Total	4112	1523	441	134	234	6444

**Fig 6:**
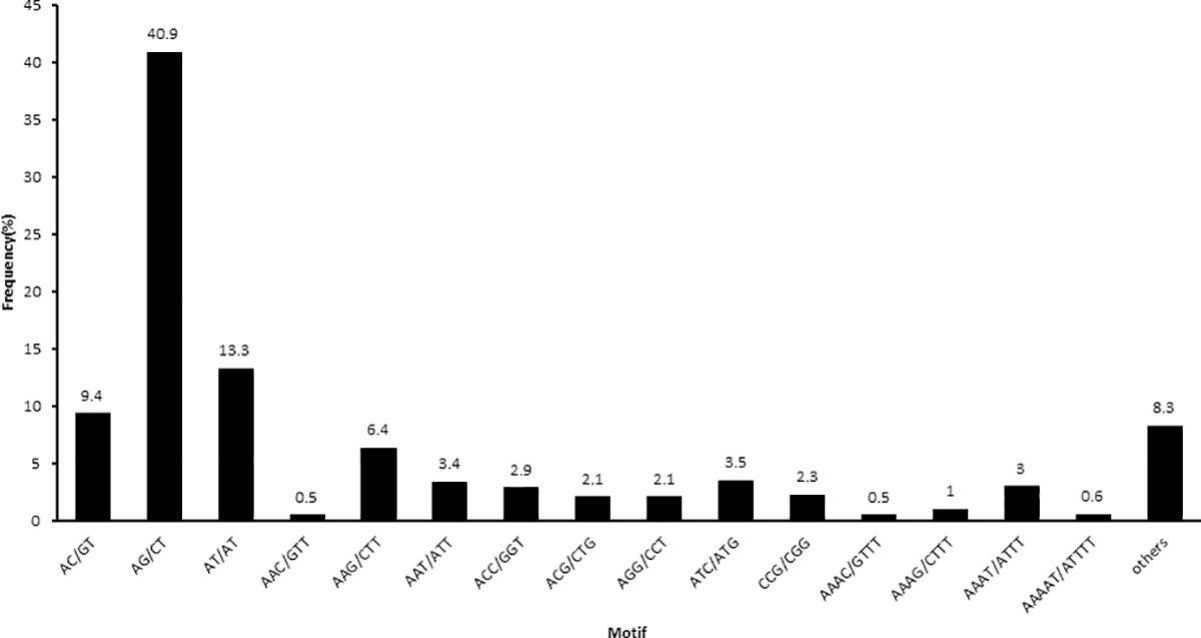
Proportion statistics of different SSR repeat types in all. The *X*-axis shows the different repeat motif types, and the percentages of the repeat motif types are shown in the *Y*-axis.

Among all SSR loci, there were 180 different repeat types. AG/CT (2,636, 40.9%) was the most abundant, followed by AT/AT (854, 13.3%) and AC/GT (604, 9.4%). Among the trinucleotide repeats, AAG/CTT (413, 6.4%) was the most abundant, followed by AAT/ATT (216, 3.4%). The AAAT/ATTT (194, 3%) motif comprised the most common tetranucleotide, and the most common pentanucleotide was AAAAT/ATTTT (38, 0.6%) (Fig 6). The repeat number of most SSRs ranged from four to fifteen, and the most frequent repeat number was six (1,729, 26.83%), followed by five (1,093, 16.96%) and seven (1,090, 16.91%). Among the dinucleotides, the most abundant number was six (1,362, 33.12%), and the most common trinucleotide and tetranucleotide number was five (935, 61.39%) and four (339, 76.41%), respectively (Table 2).

### Primer design and screening of SSR markers

In this study, 200 SSR loci were randomly selected, and primers for these loci were designed and synthesized (S1 File). Then, materials from 6 germplasm resources were used to evaluate the amplification effect, and polymorphisms were screened with 8% polyacrylamide gel electrophoresis. Among the 200 random primers, 77 (38.50%) produced expected size bands, of which 30 (38.96%) showed polymorphism (S1 Fig).

### Genetic diversity analysis

To validate the genetic diversity level of 20 *C*. *trichotomum* germplasm resources with fluorescence labeled capillary electrophoresis, we used the 30 polymorphic SSR primers mentioned above (S1 Table); 200 alleles were produced, 197 of which showed polymorphism (S2 Fig). The number of alleles (Na) ranged from 3 to 14, with an average of 6.7. The effective number of alleles (Ne) ranged from 1.4097 to 7.5850, with an average of 3.7543. Nei’s diversity index (H) ranged from 0.2907 to 0.8682, with an average of 0.6918. The Shannon information index (I) was between 0.8045 and 3.3362, with an average of 2.0825. The maximum PIC was 0.8566, and the minimum was 0.2691, with an average of 0.6504 (S2 Table).

The genetic similarity coefficient of 20 *C*. *trichotomum* was between 0.5787 and 0.9645, with an average of 0.7158, a maximum between the two populations in Peach Blossom Valley in Shandong and Pingdu in Shandong, and a minimum among Yangling, Shanxi, and Luanchuan, Henan resources. These results indicated some genetic differentiation among the 20 populations, and the genetic diversity was high.

Correlation analysis showed that H and PIC had the maximum correlation, with a coefficient of 0.9922, followed by Ne and I, and I and PIC, with correlation coefficients of 0.9679 and 0.9696, respectively. Na and H had the minimum correlation, with a correlation coefficient of 0.6662. In addition, the correlation coefficients of Ne with other indexes were all greater than those of Na with other polymorphism indexes (Table 3).

**Table 3:** Correlation coefficient between polymorphism indexes.

Index	(*Na*)	(*Ne*)	(*H*)	(*I*)	(PIC)
(*Na*)					
(*Ne*)	0.8489				
(*H*)	0.6662	0.8755			
(*I*)	0.8538	0.9679	0.9427		
(PIC)	0.7069	0.9109	0.9922	0.9676	

Na: number of alleles, Ne: effective number of alleles, H: Nei’s diversity index, I: Shannon information index, PIC: polymorphic information content.

**Fig 7:**
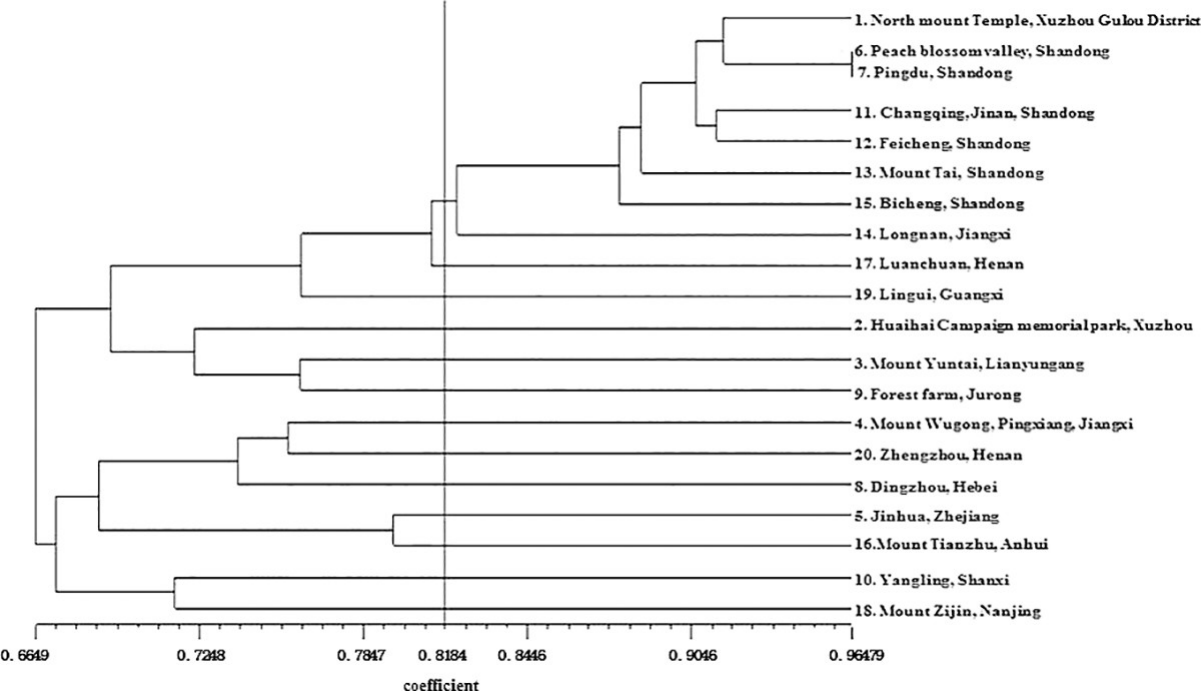
Genetic clustering diagram of the *C*. *trichotomum* germplasm lines.

According to the genetic similarity coefficients, the genetic clustering diagram of *C*. *trichotomum* was preliminary constructed through the UPGMA method. In the diagram, 20 germplasm resources could be divided into two categories when the coefficient was 0.6649. When the coefficient was 0.8184, the resources from Shandong province were all clustered together, accompanied by the populations of North Mount Temple, Xuzhou, and Longnan, Jiangxi (Fig 7).

## Discussion

*C*. *trichotomum* is an ornamental plant with various values [24, 25]. Studies on *C*. *trichotomum* have mainly focused on growth characteristics, stress resistance, chemical components, and pharmacological applications [13, 26, 27]. But the studies focusing on molecular marker development and genetic diversity are limited, and there is still no transcriptome analysis report about this species, which extremely blocks the in-depth study on *C*. *trichotomum* [15]. This study presents the first genetic research on mixed tissues of *C*. *trichotomum* through transcriptome sequencing. Notably, a large number of *C*. *trichotomum* unigenes (58,345) were generated with the Illumina HiSeq 4000 platform, and numerous ESTs were available. Among the identified unigenes, 36,900 (63.24%) were successfully annotated through BLAST searching against the public Nr, GO, COG, KOG, and KEGG databases. GO and COG analyses revealed the distribution of functional genes in this plant. KEGG database searching successfully revealed the functions of cellular-process genes and the gene products of metabolic processes. Moreover, the ‘replication, recombination and repair’, ‘signal transduction mechanisms’, and ‘transcription’ pathway were detected, which reflects the strong ability of the plant to undergo different environmental adaptations. Previous studies have identified a variety of constituents in this genus, including monoterpene, sesquiterpene, diterpenoids, triterpenoids, flavonoids and flavonoid glycosides, phenylethanoid glycosides, and steroids and steroid glycosides, in accordance with the unigene-enrichment in ‘secondary metabolite biosynthesis, transport and catabolism’. Finally, 1,720 *C*. *trichotomum* unigenes were identified within the TF families, which was larger than *Oryza sativa* L. (1611), and *Arabidopsis thaliana* (1510). Among those TF families, ‘bHLH’ possessed the most unigenes (138), followed by ‘ERF’ (133), which were similar to most plants.

RNA-sequencing is considered an effective way to acquire EST sequences for identifying novel genes and developing SSR markers [28, 29]. In this study, the overall strategy involved mixed tissue RNA-Seq together with transcriptome information technology. All 58,345 unigenes were used to detect SSRs, and finally 5,530 (9.48%) unigenes containing 6,444 SSRs were identified, which is lower than *Eucommia ulmoides* (33.99%) [30], and *Lycium barbarum* (27.93%) [31], but is higher than *Eriobotrya japonica* (6.77%) [32]. On average, the SSR loci were found every 7.70 kbp, which is lower than *Eucommia ulmoides* (0.73kbp), and *Lycium barbarum* (2.91kbp), but is higher than *Eriobotrya japonica* (14.04kbp). Compared with the 19 microsatellite loci used in the previous study, more comprehensive research was conducted in this work [15]. A larger number of SSRs results in stronger capabilities for environmental adaptation [33]. Therefore, *C*. *trichotomum* may be a good candidate for ecological restoration. Among the mined SSRs, dinucleotide motifs were the most enriched (4,112, 63.81%), followed by tri- (1,523, 23.63%) and tetra- (441, 6.84%) nucleotides. Those results were consistent with those in most plant species[34]. Compared with the previous SSR reports, the number of polymorphic primers was higher; however, the polymorphism level and PIC values of *C*. *trichotomum* were lower[35, 36]. These results may be attributed to the lower expected numbers of SSRs and polymorphisms in DNA protein-coding sequences than in non-coding sequences, and the mutation rate within these regions being lower than that in other DNA sequences.

With the development and application of molecular marker technology, research on plant resources has developed from analysis of phenotype to genotype [37]. Detecting the genetic differences at the DNA level has the advantages of individual specificity and environmental stability [38]. Molecular marker technology reflects genetic differences at the DNA level and is commonly used as a powerful means of evaluation of genetic diversity [38, 39]. Among all molecular marker technologies, SSR techniques have the advantages of widespread application, high polymorphism, and superior repeatability, and they are now broadly applied in plant breeding and genetics [17]. The genetic diversity level of 20 *C*. *trichotomum* germplasm resources was validated with fluorescence labeled capillary electrophoresis. As a result, 200 alleles were produced, 197 (98.5%) of which showed polymorphism, and the number of alleles (Na) ranged from 3 to 14, with an average of 6.7, which were similar to those of pear [40]. As reported by Mizusawa et al. [15], the number of alleles (Na) ranged from 2 to 15, with an average of 7.1 in *C*. *trichotomum*, which was similar to the result based on transcriptome in this study. Previous studies have indicated that the polymorphism level is low when 0<PIC<0.25, the polymorphic level is medium when 0.25<PIC<0.5, and the polymorphic level is high when PIC>0.5 [41]. According to the theory, 25 polymorphic loci in this study are high-level polymorphic loci, and 5 of them are medium-level polymorphic loci. These results indicate that 30 primer pairs in this study should be used in the genetic diversity analysis of *C*. *trichotomum*. The genetic diversity analysis above showed that most *C*. *trichotomum* resources clustered according to origin area, thus, indicating that geographic variation is a significant cause of germplasm variation and genetic diversity. In genetic similarity coefficient and clustering analysis, all *C*. *trichotomum* resources collected in this work were distinguished. The genetic diversity pattern of common *C*. *trichotomum* in China is probably a consequence of a complicated interaction of evolutionary forces such as environmental adaptation or ecotype differentiation.

## Conclusions

RNA sequencing is a highly effective technique for SSR identification in non-model species that lack reference genomes and transcriptomic resources. In this study, 58,345 unigenes were assembled, 36,900 of which were annotated. Then, 6,444 SSRs were detected, and 200 primer pairs were randomly selected. Finally, 30 primer pairs were successfully selected. The genetic diversity and transcription factor families were identified. These data may accelerate the identification of functional genes and genetic variations. Finally, the results of this study should provide a theoretical basis for further investigations in species classification, germplasm-resource protection, genetic diversity analysis, and molecular marker assisted breeding in *C*. *trichotomum*.

## Supporting information

S1 Fig Representative polyacrylamide gel electrophoresis pictures. M: marker; 33–48, 81–96: primer 33–48, primer 81–96.
(TIF)

S2 Fig Representative fluorescence-labeled capillary electrophoresis pictures. This figure presents part of polymorphism idetification results with primer 87 in the 20 *C*. *trichotomum* popolations. A: Pingxiang, Jiangxi (PX), B: Jinhua, Zhejiang (JH), C: Tai’an, Shandong (TA).
(TIF)

S1 Table Characteristics of 30 polymorphic SSR markers of *C*. *trichotomum*. (DOC)

S2 Table Polymorphism detection of 30 primers in 20 *C*. *trichotomum* germplasm resources. Na: number of alleles, Ne: effective number of alleles, H: Nei’s diversity index, I: Shannon information index, PIC: polymorphic information content.
(DOCX)

S1 File The complete list of the identified primer pairs in this study. (XLS)
