# Supplementary figures and images for: SSR marker development in Clerodendrum trichotomum using transcriptome sequencing
Source: PLoS One. 2019 Nov 20;14(11):e0225451. doi: 10.1371/journal.pone.0225451 (PMC6867647; doi:10.1371/journal.pone.0225451)

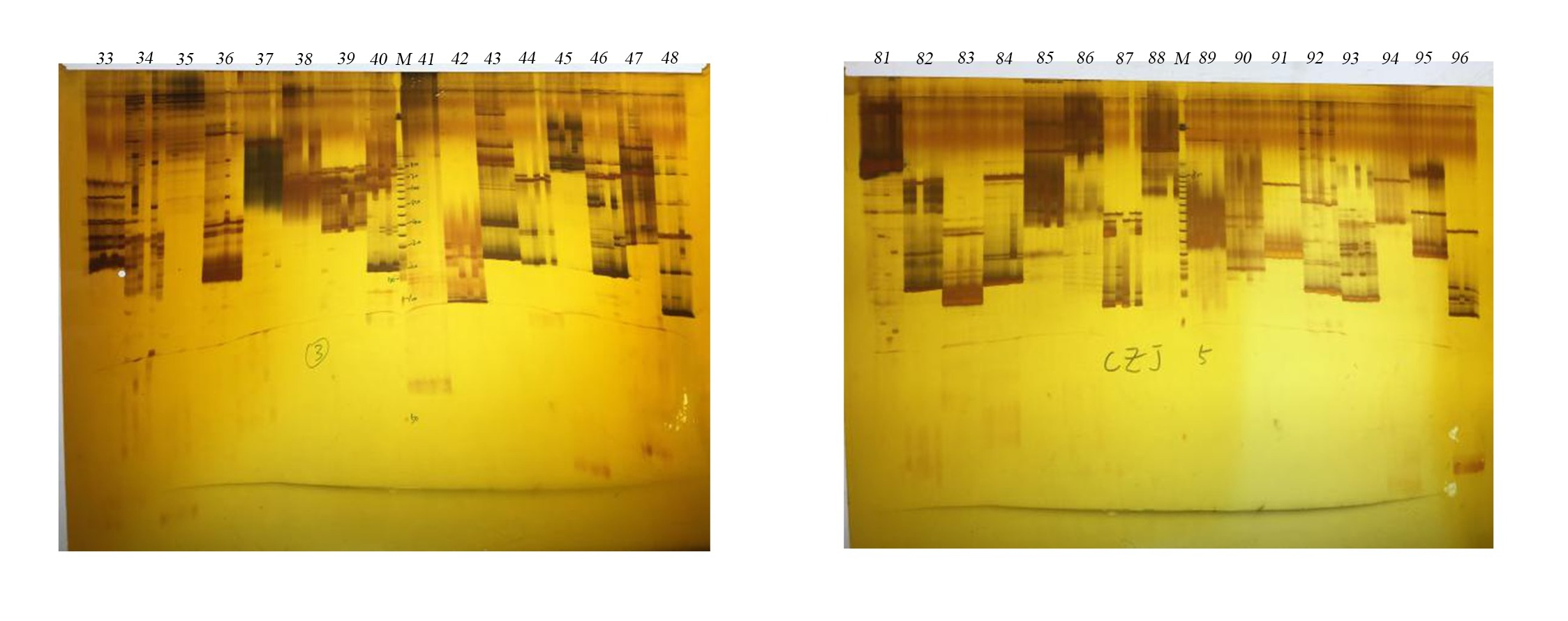

Supplement: S1 Fig — M: marker; 33–48, 81–96: primer 33–48, primer 81–96. (TIF) [file pone.0225451.s001.tif]

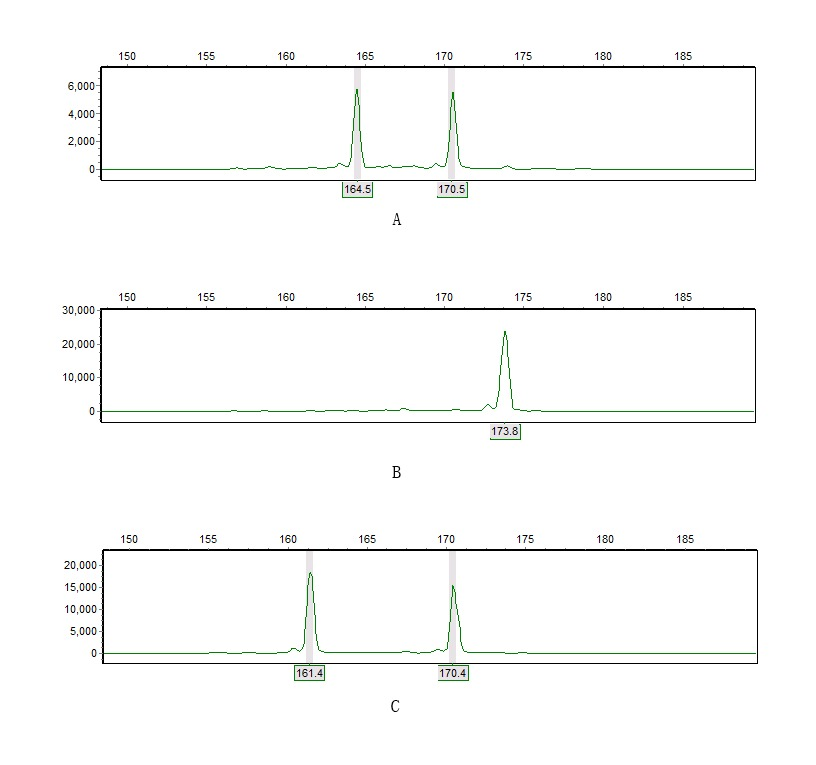

Supplement: S2 Fig — This figure presents part of polymorphism idetification results with primer 87 in the 20 C. trichotomum popolations. A: Pingxiang, Jiangxi (PX), B: Jinhua, Zhejiang (JH), C: Tai’an, Shandong (TA). (TIF) [file pone.0225451.s002.tif]
