# Supplementary material for: SSR marker development in Clerodendrum trichotomum using transcriptome sequencing
Source: PLoS One. 2019 Nov 20;14(11):e0225451. doi: 10.1371/journal.pone.0225451 (PMC6867647; doi:10.1371/journal.pone.0225451)
Supplement: S1 Table — (DOC) [file pone.0225451.s003.doc]

**S1 Table. Characteristics of 30 polymorphic SSR markers of *C. trichotomum.***

| ID | Unigene | SSR loci | Primer sequence  （5’→3’） | tTa (℃) | Target fragment length（bp） | Putative function [organism] |
| --- | --- | --- | --- | --- | --- | --- |
| 13 | Unigene0000308 | (CT)9 | F:TTCCTCACCTTTGCACTTGA  R:GCCATTTTGCAATTTTTGCT | 60 | 198 | type I inositol 1,4,5-trisphosphate 5-phosphatase CVP2-like isoform X2 [*Sesamum indicum*] |
| 31 | Unigene0001149 | (ATT)6 | F:GCAGTTGGAGGATGGTAGGA  R:TACCAAAAATTCGACTCGCC | 60 | 220 | ubiquitin 11 [*Arabidopsis thaliana*] |
| 33 | Unigene0001354 | (CT)8 | F:TCCACCAATTAGGGTTTCCA  R:TAGCAAAGGCTCGTTTTGGT | 60 | 183 | transcription factor bHLH68-like isoform X1 [*Sesamum indicum*] |
| 39 | Unigene0002238 | (GAA)5 | F:TTGGGGAAACAGATCTACCG  R:CCCCTGTGATCAACACCTTT | 60 | 234 | probable galactinol--sucrose galactosyltransferase 6 [*Sesamum indicum*] |
| 44 | Unigene0001681 | (GA)10 | F:TGCAGAACAAACATCCCGTA  R:TGATTATTTTTCCCGCTTGC | 60 | 158 | unknown |
| 46 | Unigene0002723 | (CCG)5 | F:GTTCTATTCGCATAAGCGGC  R:AACCAAATCAGGATCCTCC | 60 | 232 | mastermind-like protein 2 [*Malus domestica*] |
| 57 | Unigene0003426 | (CAG)5 | F:ACATCGAAAGAAAACCCACG  R:AAAGTAGGTTGCAGAGGGGG | 60 | 108 | leucine-rich repeat extensin-like protein 4 [*Sesamum indicum*] |
| 61 | Unigene0003623 | (TGA)8 | F:TTTGGGGTTGATCTGACACA  R:TCTGTGCCTCTAAGCCTCGT | 60 | 162 | DNA ligase 1-like [*Sesamum indicum*] |
| 68 | Unigene0003997 | (TTG)6 | F:TTCGTGGCTTGCTTTGATAA  R:CGAAAAGGCATAGCAACAAGA | 60 | 224 | unknown |
| 75 | Unigene0004410 | (CT)10 | F:GGGCTCACACCATAAATTCG  R:CATTTGGACCTTCCCTCTCA | 60 | 215 | MADS-box protein SOC1-like isoform X1 [*Sesamum indicum*] |
| 78 | Unigene0004638 | (GA)7 | F:ACAAGGAGCGATTGGTGATA  R:GCAAGATTATCGGGAGGTGA | 60 | 161 | unknown |
| 87 | Unigene0005727 | (GAT)5 | F:GGAACAGCAGAAGCGATACC  R:GTTGGGAGCTTGATGTTTCC | 60 | 171 | probably inactive leucine-rich repeat receptor-like protein kinase At5g48380 [*Sesamum indicum*] |
| 93 | Unigene0006463 | (GGT)8 | F:GAGCGAACCTTGATAGCTGC  R:ATCAATTCAAAGCCCCTGT | 60 | 167 | Adenine nucleotide alpha hydrolases-like superfamily protein isoform 2 [*Theobroma cacao*] |
| 96 | Unigene0006726 | (AG)6 | F:ATTGGGGCAAATTGACAGAG  R:TCGCAGCCGTTAAATAGTC | 60 | 166 | transcription factor HBP-1a-like isoform X2 [*Sesamum indicum*] |
| 99 | Unigene0003439 | (CCA)10 | F:TCCTCCTCTACAAGGCGAAA  R:TTCTGCTCGAAGATCGTGAA | 60 | 191 | unknown |
| 113 | Unigene0030389 | (AG)7 | F:GGGTTCAAAATCTTGCCAAA  R:TGGCACCTACAGCAAACAAG | 60 | 139 | auxin-responsive protein IAA27 [*Sesamum indicum*] |
| 115 | Unigene0030544 | (TGA)6 | F:GCAATCCTCGGATCATTGTT  R:CTCACCAGAGAGACGGTTCA | 60 | 239 | nicotinamide adenine dinucleotide transporter 1, chloroplastic [*Nicotiana tomentosiformis*] |
| 116 | Unigene0036814 | (AAG)5 | F:TCGTGGTTCAGCGATCTGTA  R:GCTGAGGATCTTTCACGGAG | 60 | 195 | unknown |
| 118 | Unigene0036910 | (AT)9 | F:CACGAACCATTCATTCTGCT  R:GTTGATTTGTTCGGTTTGGG | 60 | 206 | AT-hook motif nuclear-localized protein 25-like [*Jatropha curcas*] |
| 121 | Unigene0037074 | (CT)7 | F:GCAAGAAAACCCCATTGAAA  R:AAACCTTCAACTGCCCCTCT | 60 | 173 | zinc finger protein NUTCRACKER-like, partial [*Sesamum indicum*] |
| 130 | Unigene0037668 | (TA)9 | F:GCATAATCATCATGAAGAGAAAACA  R:TCTGGAGGAGTCCCTCTCAA | 60 | 191 | BnaAnng09550D [*Brassica napus*] |
| 131 | Unigene0037782 | (AAAC)6 | F:GTTGCTGAGGATTGAGGAGG  R:CCCACCAAAACACGGTAAGT | 60 | 178 | enolase [*Sesamum indicum*] |
| 135 | Unigene0038100 | (CAGCCT)4 | F:TCCACTATCGCATCCAATGA  R:AATACGGAAACAGCCAATGC | 60 | 254 | histone-lysine N-methyltransferase, H3 lysine-9 specific SUVH6-like isoform X2 [*Nicotiana sylvestris*] |
| 142 | Unigene0038706 | (AG)6 | F:TGATCCCAGGTACGGAAATC  R:ATCACTCCGTCACCTCCATC | 60 | 235 | probable alpha,alpha-trehalose-phosphate synthase [UDP-forming] 11 [*Sesamum indicum*] |
| 171 | Unigene0041777 | (CTC)5 | F:CTGCTGCAGTTGCAACACTT  R:ACCGATCTCGTTCCTTGATG | 60 | 214 | integral membrane protein [*Triticum urartu*] |
| 175 | Unigene0042149 | (GA)7 | F:TGGTTAAAGGCTGCTTGCTT  R:AGCCAAAATAGCTCACCGAA | 60 | 161 | homeobox-leucine zipper protein REVOLUTA-like [*Sesamum indicum*] |
| 179 | Unigene0042482 | (TA)7 | F:TGCCACAAAGCAGCAGTTAC  R:CCATACCCAAGAAGAGGCAA | 60 | 239 | pyrophosphate-energized membrane proton pump 3 [*Sesamum indicum*] |
| 182 | Unigene0042839 | (GCT)5 | F:AGGGTCATCCAAGATCGACA  R:TCAGCCATCTCTGTCAAACG | 60 | 157 | chaperone protein ClpB1-like [*Glycine max*] |
| 183 | Unigene0043038 | (AG)21 | F:CCACTGCTTTTCCCACTTTC  R:TTGTTCGCACTCTCAACCTG | 60 | 168 | receptor-like protein kinase BRI1-like 3 [*Sesamum indicum*] |
| 193 | Unigene0050170 | (AT)8 | F:CCAAACAAGTGGCCAAACTAA  R:CCATAATGCATCATCCAACCT | 60 | 120 | gag protein [*Populus trichocarpa*] |
