# Supplementary material for: SSR marker development in Clerodendrum trichotomum using transcriptome sequencing
Source: PLoS One. 2019 Nov 20;14(11):e0225451. doi: 10.1371/journal.pone.0225451 (PMC6867647; doi:10.1371/journal.pone.0225451)
Supplement: S2 Table — Na: number of alleles, Ne: effective number of alleles, H: Nei’s diversity index, I: Shannon information index, PIC: polymorphic information content. (DOCX) [file pone.0225451.s004.docx]

**S2 Table. Polymorphism detection of 30 primers in 20 *C. trichotomum* germplasm resources.** Na: number of alleles, Ne: effective number of alleles, H: Nei’s diversity index, I: Shannon information index, PIC: polymorphic information content.

| Primer code | Allele size range (bp) | | (Na) | (Ne) | (H) | (I) | (PIC) |
| --- | --- | --- | --- | --- | --- | --- | --- |
| 13 | 288-326 | | 14 | 7.5850 | 0.8682 | 3.3362 | 0.8566 |
| 31 | 198-223 | | 8 | 4.6093 | 0.7830 | 2.4953 | 0.7525 |
| 33 | 179-188 | | 4 | 2.3469 | 0.5739 | 1.4997 | 0.5197 |
| 39 | 231-254 | | 5 | 3.6789 | 0.7282 | 2.0518 | 0.6843 |
| 44 | 204-225 | | 10 | 4.4347 | 0.7745 | 2.5751 | 0.7441 |
| 46 | 225-240 | | 7 | 5.1867 | 0.8072 | 2.5655 | 0.7824 |
| 57 | 104-113 | | 3 | 1.4260 | 0.2987 | 0.8071 | 0.2727 |
| 61 | 152-167 | | 6 | 3.9825 | 0.7489 | 2.2281 | 0.7118 |
| 68 | 220-234 | | 5 | 2.6175 | 0.6180 | 1.6635 | 0.5539 |
| 75 | 213-230 | | 9 | 4.7059 | 0.7875 | 2.6162 | 0.7636 |
| 78 | 143-161 | | 6 | 2.3131 | 0.5677 | 1.4155 | 0.4735 |
| 87 | 161-177 | | 7 | 3.2751 | 0.6947 | 2.0933 | 0.6491 |
| 93 | 160-186 | | 9 | 5.1145 | 0.8045 | 2.6817 | 0.7794 |
| 96 | 160-182 | | 6 | 2.3100 | 0.5671 | 1.6267 | 0.5279 |
| 99 | 175-184 | | 4 | 2.3738 | 0.5787 | 1.4378 | 0.4946 |
| 113 | 132-142 | | 5 | 3.7736 | 0.7350 | 2.0345 | 0.6883 |
| 115 | 238-251 | | 5 | 3.4425 | 0.7095 | 1.9810 | 0.6593 |
| 116 | 186-211 | | 6 | 3.8795 | 0.7422 | 2.1666 | 0.7002 |
| 118 | 200-214 | 8 | | 4.5977 | 0.7825 | 2.4981 | 0.7535 |
| 121 | 171-185 | 6 | | 4.2795 | 0.7663 | 2.2361 | 0.7278 |
| 130 | 170-195 | 8 | | 5.4133 | 0.8153 | 2.6346 | 0.7902 |
| 131 | 160-179 | 5 | | 2.5383 | 0.6060 | 1.6192 | 0.5358 |
| 135 | 246-269 | 5 | | 3.6081 | 0.7228 | 2.0543 | 0.6808 |
| 142 | 225-275 | 8 | | 4.4742 | 0.7765 | 2.4036 | 0.7419 |
| 171 | 200-233 | 6 | | 3.9330 | 0.7457 | 2.1839 | 0.7051 |
| 175 | 142-169 | 7 | | 2.8121 | 0.6444 | 1.9428 | 0.6059 |
| 179 | 237-246 | 4 | | 3.3796 | 0.7041 | 1.8586 | 0.6495 |
| 182 | 146-163 | 4 | | 1.4097 | 0.2907 | 0.8045 | 0.2691 |
| 183 | 214-311 | 13 | | 6.0179 | 0.8338 | 2.9687 | 0.8140 |
| 193 | 109-133 | 7 | | 3.1087 | 0.6783 | 1.9940 | 0.6256 |
| Average |  | 6.7 | | 3.7543 | 0.6918 | 2.0825 | 0.6504 |
| Variation coefficient (%) |  | 37.85 | | 36.24 | 19.71 | 27.40 | 21.99 |
